# Supplementary material for: Trophic eggs affect caste determination in the ant Pogonomyrmex rugosus
Source: eLife. 2025 Jul 31;12:RP86899. doi: 10.7554/eLife.86899 (PMC12313230; doi:10.7554/eLife.86899)
Supplement: Supplementary file 1. [file elife-86899-supp1.docx]

Trophic eggs affect caste determination in the ant *Pogonomyrmex rugosus*

E. Genzoni, T. Schwander and L. Keller

Supplementary File 1

**Supplementary File1, Table 1.** List of ant species for which previous studies described the presence of trophic eggs (T-eggs). Information is given on whether they are laid by the queen or the workers, or whether no trophic eggs were detected. na indicates that no information is available. ^1^ Possibly laid by queens at the founding stage, ^2^ only virgin queens.

| **Subfamily** | **Species** | **T-eggs laid by queen** | **T-eggs laid by worker** | **References** |
| --- | --- | --- | --- | --- |
| **Amblyoponinae** | *Amblyopone silvestrii* | No | No | (Masuko 2003) |
|  | *Amblyopone sp. (Reclinata group)* | na | Yes | (Ito 1991, 1993) |
|  | *Prionopelta kraepelini* | na | Yes | (Masuko 2003) |
| **Dolichoderinae** | *Dolichoderus quadripunctatus* | na | Yes | (Torossian 1968; Fletcher and Ross 1985) |
|  | *Linepithema humile* | na | Yes | (Bartels 1988) |
|  | *Technomyrmex albipes* | Yes | Yes | (Yamauchi *et al.* 1991) |
| **Ectatomminae** | *Ectatomma tuberculatum* | na | Yes | (Hora *et al.* 2007; Azevedo *et al.* 2011) |
|  | *Gnamptogenys menadensis* | No | Yes | (Gobin *et al.* 1998, 1999) |
|  | *Gnamptogenys costata* | na | Yes | (Gobin *et al.* 1998) |
|  | *Gnamptogenys dammemani* | na | Yes | (Gobin *et al.* 1998) |
|  | *Gnamptogenys moelleri* | na | Yes | (Gobin *et al.* 1998) |
| **Formicinae** | *Anoplolepis gracilipes* | na | Yes | (Lee *et al.* 2017) |
|  | *Cataglyphis floricola* | na | Yes | (Amor *et al.* 2017) |
|  | *Cataglyphis tartessica* | na | Yes | (Amor *et al.* 2017) |
|  | *Formica pergandei* | na | Yes | (Hung 1973) |
|  | *Lasius niger* | Possible^1^ | Yes | (Baroni Urbani 1991; Khila and Abouheif 2008) |
|  | *Oecophylla longinoda* | na | Yes | (Hölldobler and Wilson 1983; Fletcher and Ross 1985) |
|  | *Plagiolepis pygmaea* | no | Yes | (Passera 1978, 1980; Fletcher and Ross 1985) |
| **Myrmeciinae** | *Myrmecia forceps* | Yes | Yes | (Freeland 1958) |
|  | *Myrmecia gulosa* | na | Yes | (Freeland 1958; Dietemann et al. 2002) |
| **Myrmicinae** | *Pheidole pallidula* | Yes^2^ | No | (Passera 1978; Lorber and Passera 1981; Bartels 1988) |
|  | *Solenopsis invicta* | Yes | No | (Fletcher and Ross 1985; Voss et al. 1988; Cassill 2002) |
|  | *Pogonomyrmex badius* | na | na | (Freeland 1958; Smith et al. 2007) |
|  | *Pogonomyrmex barbatus* | Yes | na | (Volny et al. 2006; Smith et al. 2007) |
|  | *Pogonomyrmex J lineages* | Yes | na | (Helms Cahan *et al.* 2011) |
|  | *Acromyrmex sp.* | No | Yes | (Dijkstra et al. 2005) |
|  | *Aphaenogaster (=Novomessor) cockerelli* | na | Yes | (Hölldobler and Carlin 1989; Smith et al. 2008) |
|  | *Aphaenogaster rudis* | na | Yes | (Khila and Abouheif 2008, 2010) |
|  | *Aphaenogaster subterranea* | na | Yes | (Passera 1978) |
|  | *Atta laevigata* | Yes | Yes | (Passera 1978) |
|  | *Leptothorax acervorum* | Unlikely | Yes | (Ito 2005) |
|  | *Messor capitatus* | Unlikely | Yes | (Passera 1978; Baroni Urbani 1991) |
|  | *Messor semirufus* | Unlikely | Yes | (Baroni Urbani 1991) |
|  | *Myrmica americana* | na | Yes | (Khila and Abouheif 2008) |
|  | *Temnothorax recedens (Nyl.)* | No | Yes | (Dejean and Passera 1974) |
|  | *Zacryptocerus varians (fr. smith)* | na | Yes | (Wilson 1976) |
|  | *Acanthomyrmex careoscrobis Moffett* | Yes, by ergatoid queen | Yes | (Yamada et al. 2018) |
|  | *Acanthomyrmex ferox* | Yes | Yes | (Gobin and Ito 2000) |
|  | *Atta sp.* | na | Yes | (Dijkstra et al. 2005) |
|  | *Atta sexdens* | Yes | Yes | (Della Lucia *et al.* 1990; Augustin *et al.* 2011) |
|  | *Crematogaster smithi Creighton* | Yes | Yes | (Heinze et al. 1995, 1999) |
|  | *Myrmica rubra* | Yes | Yes | (Brian and Rigby 1978; Smeeton 1981; Wardlaw and Elmes 1995, 1998) |
|  | *Myrmica ruginodis* | Yes | Yes | (Wardlaw and Elmes 1995, 1998) |
|  | *Myrmica schencki* | Yes | Yes | (Wardlaw and Elmes 1995, 1998) |
|  | *Myrmica sulcinodis* | Yes | Yes | (Wardlaw and Elmes 1995, 1998) |
| **Nothomyrmeciinae** | *Nothomyrmecia macrops* | na | Yes | (Taylor 1978) |
| **Ponerinae** | *Hypoponera eduardi* | na | No | (Choe 1988) |
|  | *Odontomachus haematodes* | na | No | (Colombel 1972) |
|  | *Rhytidoponera purpurea* | na | na | (Haskins and Whelden 1965) |
|  | *Pachycondyla apicalis* | No | Yes | (Dietemann and Peeters 2000) |
|  | *Neoponera villosa* | na | Yes | (CamargoMathias and Caetano 1995) |
|  | *Pachycondyla krugeri* | na | Yes | (Gobin *et al.* 1998) |
|  | *Paraponera clavata* | Unlikely | Yes | (Peeters 2017) |
|  | *Diacamma rugosum* | No queen | na | (Wheeler and Chapman 1922) |

**Supplementary File 1, Table 2**. Number of worker and queen pupae that developed in recipient colonies without trophic eggs (W) or with three trophic eggs (3T). Empty cells mean that no pupae developed in this recipient colony.

**References**

Amor F, Ortega P, Boulay R, Cerdá X. 2017. Frequent colony orphaning triggers the production of replacement queens via worker thelytoky in a desert-dwelling ant. *Insectes Sociaux* **64**:373–378. 10.1007/s00040-017-0556-9.

Augustin JO, Santos JF, Elliot SL. 2011. A behavioral repertoire of Atta sexdens (Hymenoptera, Formicidae) queens during the claustral founding and ergonomic stages. *Insectes Sociaux* **58**:197–206. 10.1007/s00040-010-0137-7.

Azevedo DO, Zanuncio JC, Delabie JHC, Serrão JE. 2011. Temporal variation of vitellogenin synthesis in Ectatomma tuberculatum (Formicidae: Ectatomminae) workers. *Journal of Insect Physiology* **57**:972–977. 10.1016/j.jinsphys.2011.04.015.

Baroni Urbani CB. 1991. Indiscriminate oophagy by ant larvae: an explanation for brood serial organization. *Insectes Sociaux* **38**:229–239. 10.1007/BF01314909.

Brian MV, Rigby C. 1978. The trophic eggs of Myrmica rubra L. *Insectes Sociaux* **25**:89–110. 10.1007/BF02224488.

Camargo MM, Caetano F. 1995. Trophic eggs in workers of Neoponera villosa ants (Hymenoptera:Ponerinae). *J. Adv. Zool* **16**:62–66.

Cameron RC, Duncan EJ, Dearden PK. 2013. Biased gene expression in early honeybee larval development. *BMC Genomics* **14**:1–12. 10.1186/1471-2164-14-903

Cassill D. 2002. Brood care strategies by newly mated monogyne Solenopsis invicta (Hymenoptera: Formicidae) queens during colony founding. *Annals of the Entomological Society of America* **95**:208–212. 10.1603/0013-8746(2002)095[0208:BCSBNM]2.0.CO;2.

Choe JC. 1988. Worker reproduction and social evolution in ants (hymenoptera: formicidae). *Advances in Myrmecology*. E. J. Brill. p. 163–187. 10.1163/9789004630765_014.

Collins DH, Mohorianu I, Beckers M, Moulton V, Dalmay T, Bourke AF. 2017. MicroRNAs associated with caste determination and determination in a primitively eusocial inAssociated with Caste Determination and Determination in a Primitively Eusocial Insect. *Scientific Reports* **7**:1–9. 10.1038/srep45674.

Della Lucia TMC, Vilela EF, Moreira DDO, Bento JMS, Anjos ND. 1990. Egg laying in atta sexdens rubropilosa, under laboratory conditions. *Applied Myrmecology: A World Perspective*. Westview Press. p. 173–179.

Dijkstra MB, Nash DR, Boomsma JJ. 2005. Self-restraint and sterility in workers of Acromyrmex and Atta leafcutter ants. *Insectes Sociaux* **52**:67–76. 10.1007/s00040-004-0775-8.

Fletcher DJC, Ross KG. 1985. Regulation of reproduction in eusocial hyReproduction in Eusocial Hymenoptera. *Annual Review of Entomology* **30**:319–343. 10.1146/annurev.en.30.010185.001535

Freeland J. 1958. Biological and social patterns in the Australian bulldog ants of the genus Myrmecia. *Australian Journal of Zoology* **6**:1–18. 10.1071/ZO9580001.

Gobin B, Billen J, Peeters C. 1999. Policing behaviour towards virgin egg layers in a polygynous ponerine ant. *Animal Behaviour* **58**:1117–1122. 10.1006/anbe.1999.1245.

Haskins CP, Whelden RM. 1965. “Queenlessness,” worker sibship, and colony versus population structure in the formicid genus *Rhytidoponera*. *Psyche* **72**:87–112. 10.1155/1965/40465.

Hölldobler B, Wilson EO. 1983. Queen control in colonies of weaver ants (hymenoptera: fControl in Colonies of Weaver Ants (Hymenoptera: Formicidae). *Annals of the Entomological Society of America* **76**:235–238. 10.1093/aesa/76.2.235.

Hölldobler B, Carlin NF. 1989. Colony Founding, Queen Control and Worker Reproduction in the Ant *Aphaenogaster (=Novomessor) Cockerelli* (Hymenoptera: Formicidae). *Psyche* **96**:131–151. 10.1155/1989/74135.

Hora RR, Poteaux C, Doums C, Fresneau D, Fénéron R. 2007. Egg cannibalism in a facultative polygynous ant: Conflict for reproduction or strategy to survive. *Ethology : Formerly Zeitschrift Fur Tierpsychologie* **113**:909–916. 10.1111/j.1439-0310.2007.01391.x.

Hung ACF. 1973. Reproductive biology in dulotic ants: preliminary report (Hymenoptera: Formicidae). *Entomological News* **84**:253–259.

Ito F. 1991. Preliminary report on queenless reproduction in a primitive ponerine ant Amblypone sp. (reclinata group) in West Java, Indonesia. *Psyche* **98**:319–322.

Ito F. 1993. Social organization in a primitive ponerine ant: Queenless reproduction, dominance hierarchy and functional polygyny in Amblyopone sp. *J. Nat. Hist* **27**:1315–1324. 10.1080/00222939300770751.

Ito F. 2005. Mechanisms regulating functional monogyny in a Japanese population of Leptothorax acervorum (Hymenoptera, Formicidae): Dominance hierarchy and preferential egg cannibalism. *Belgian J. Zool* **135**:3–8.

Khila A, Abouheif E. 2008. Reproductive constraint is a developmental mechanism that maintains social harmony in advanced ant societies. *PNAS* **105**:17884–17889. 10.1073/pnas.0807351105.

Khila A, Abouheif E. 2010. Evaluating the role of reproductive constraints in ant social evolution. *Philos. Trans. R. Soc. B* **365**:617–630. 10.1098/rstb.2009.0257.

Klowden MJ. 2013. Developmental systems. In: Klowden MJ (Ed). *Physiological Systems in Insects*. (3rd ed.). Academic Press. p. 149–196.

Masuko K. 2003. Larval oophagy in the ant Amblyopone silvestrii (Hymenoptera, Formicidae). *Insectes Sociaux* **50**:317–322. 10.1007/s00040-003-0688-y.

Passera L. 1978. Une nouvelle catégorie d’œufs alimentaires: les œufs alimentaires émis par les reines vierges depheidole pallidula (Nyl.) (Formicidae, Myrmicinae). *Insectes Sociaux* **25**:117–126. 10.1007/BF02224251.

Peeters C. 2017. Independent colony foundation in Paraponera clavata (hymenoptera: Formicidae): First workers lay trophic eggs to feed queen’s larvae. *Sociobiology* **64**:417–422. 10.13102/sociobiology.v64i4.2092.

Smeeton L. 1981. The source of males in Myrmica rubra L. (Hym. Formicidae). *Insectes Sociaux* **28**:263–278. 10.1007/BF02223628.

Smith CR, Schoenick C, Anderson KE, Gadau J, Suarez AV. 2007. Potential and realized reproduction by different worker castes in queen-less and queen-right colonies of Pogonomyrmex badius. *Insect. Soc* **54**:260–267. 10.1007/s00040-007-0940-y.

Smith AA, Hölldobler B, Liebig J. 2008. Hydrocarbon signals explain the pattern of worker and egg policing in the ant Aphaenogaster cockerelli. *Journal of Chemical Ecology* **34**:1275–1282. 10.1007/s10886-008-9529-9.

Søvik E, Bloch G, Ben-Shahar Y. 2015. Function and evolution of microRNAs in eusocial Hymenoptera. *Frontiers in Genetics* **6**:1–11. 10.3389/fgene.2015.00193.

Suzzoni JP, Passera L, Strambi A. 1979. *Taux Des Ecdystéroides et Déterminisme Des Castes de La Fourmi Pheidole Pallidula (Hym. Formicidae)*.

Taylor RW. 1978. Nothomyrmecia macrops: a living-fossil ant rea Macrops: A Living-Fossil Ant Rediscovered. *Science* **201**:979–985. 10.1126/science.201.4360.979.

Torossian C. 1968. Recherches sur la biologie et l’éthologie deDolichoderus Quadripunctatus (L) (Hym. Form. Dolichoderidæ). *Insectes Sociaux* **15**:375–387. 10.1007/BF02223634.

Volny VP, Greene MJ, Gordon DM, Volny VP, Greene MJ, Gordon DM. 2006. Brood production and lineage discrimination in the red harvester ant (Pogonomyrmex barbatus). *Ecology* **87**:2194–2200. 10.1890/0012-9658(2006)87[2194:bpaldi]2.0.co;2, 16995618.

Voss SH, McDonald JF, Keith CH. 1988. Production and abortive development of fire ant trophic eggs. In: Trager JC (Ed). *Advances in Myrmecology*. E. J. Brill. p. 517–534.

Wardlaw JC, Elmes GW. 1998. Variability in oviposition by workers of six species of Myrmica (hymenoptera, formicidae). *Insectes Sociaux* **45**:369–384. 10.1007/s000400050096.

Wheeler WM. 1910. *Ants: Their Structure, Development, and Behavior*. Columbia University Press.

Yamada A, Ito F, Hashim R, Eguchi K. 2018. Queen polymorphism in Acanthomyrmex careoscrobis Moffett, 1986 in Peninsular Malaysia (Hymenoptera: Formicidae: Myrmicinae), with descriptions of hitherto unknown female castes and males. *Asian Myrmecology* **10**:1–19. 10.20362/am.010009.

Yamauchi K, Furukawa T, Kinomura K, Takamine H, Tsuji K. 1991. Secondary polygyny by inbred wingless sexuals in the dolichoderine ant Technomyrmex albipes. *Behavioral Ecology and Sociobiology* **29**:313–319. 10.1007/BF00165955.
